# Neuroprotection by the Traditional Chinese Medicine, Tao-Hong-Si-Wu-Tang, against Middle Cerebral Artery Occlusion-Induced Cerebral Ischemia in Rats

**DOI:** 10.1155/2011/803015

**Published:** 2010-11-04

**Authors:** Chih-Jen Wu, Jui-Tai Chen, Ting-Lin Yen, Thanasekaran Jayakumar, Duen-Suey Chou, George Hsiao, Joen-Rong Sheu

**Affiliations:** ^1^Division of Nephrology, Mackay Memorial Hospital, Taipei 104, Taiwan; ^2^Mackay Medicine, Nursing and Management College, Taipei 112, Taiwan; ^3^Department of Pharmacology, School of Medicine, College of Medicine, Taipei Medical University, 250 Wuxing St., Taiwan; ^4^Department of Anesthesiology, WanFang Hospital, and Graduate Institute of Clinical Medicine, Taipei Medical University, Taipei 110, Taiwan

## Abstract

Tao-Hong-Si-Wu-Tang (THSWT) is a famous traditional Chinese medicine (TMC). In the present study, oral administration of THSWT (0.7 and 1.4 g kg^−1^day^−1^) for 14 days before MCAO dose-dependently attenuated focal cerebral ischemia in rats. MCAO-induced focal cerebral ischemia was associated with increases in hypoxia-inducible factor (HIF)-1*α*, inducible nitric oxide synthase (iNOS), tumor necrosis factor (TNF)-*α*, and active caspase-3 expressions in ischemic regions. These expressions were obviously inhibited by 0.7 g kg^−1^day^−1^ THSWT treatment. In addition, THSWT inhibited platelet aggregation stimulated by collagen in washed platelets. In an *in vivo* study, THSWT (16 g kg^−1^) significantly prolonged platelet plug formation in mice. However, THSWT (20 and 40 *μ*g mL^−1^) did not significantly reduce the electron spin resonance (ESR) signal intensity of hydroxyl radical (OH^•^) formation. In conclusion, the most important findings of this study demonstrate for the first time that THSWT possesses potent neuroprotective activity against MCAO-induced focal cerebral ischemia *in vivo*. This effect may be mediated, at least in part, by the inhibition of both HIF-1*α* and TNF-*α* activation, followed by the inhibition of inflammatory responses (i.e., iNOS expression), apoptosis formation (active caspase-3), and platelet activation, resulting in a reduction in the infarct volume in ischemia-reperfusion brain injury.

## 1. Introduction

Ischemic brain injury often causes irreversible brain damage. The cascade of events leading to neuronal injury and death in ischemia includes the release of cytokines, free radicals, and platelet activation [1, 2]. Reperfusion of ischemic areas can exacerbate ischemic brain damage through the generation of reactive oxygen species (ROS) including superoxide anions (O_2_^·−^), hydroxyl radicals (OH^·^), and nitric oxide (NO) [3, 4]. Leukocytes are a potential source of ROS when activated during inflammatory responses [5]. When a tissue suffers from ischemia and reperfusion, proinflammatory cytokines produced by inflammatory cells can trigger adhesion and migration of circulating leukocytes to endothelial cells and generation of ROS that enhances ischemic injury [6]. Furthermore, ROS also mediate a mitochondrial signaling pathway that may lead to apoptosis followed by cerebral ischemia [4]. Therefore, both the inhibition of production and enhanced degradation of ROS by pharmacological agents were found to limit the extent of brain damage following stroke-like events [7]. Furthermore, the participation of activated platelets was observed in brain microvessels of the ischemic microvascular bed after experimental middle cerebral artery occlusion (MCAO) [2]. Microvascular thrombi continue to accumulate even after recanalization of the MCAO, contributing to postischemic hypoperfusion and ongoing neuronal damage [8]. Thus, platelet aggregation may play a crucial role in MCAO-induced cerebral damage.

Traditional Chinese medicines (TCMs) have successfully been used for centuries to treat a wide variety of ailments and have attracted increasing attention from industry and academia in China [9, 10]. However, their therapeutic mechanisms and effects are still not well understood. Nowadays, it is widely accepted that multiple ingredients are responsible for the therapeutic effects of TMCs. For example, Tao-Hong-Si-Wu-Tang (THSWT) is a famous TMC formula for treating cardiovascular diseases (CVDs) with a history of several centuries. The formula mainly consists of six plant materials: *Shu Di Huang (Rehmannia glutinosa *Liboschitz)*, Bai Shao (Paeonia lactiflora *Pallas*), Dang Gui (Angelica sinensis *(Oliv.) Diels)*, Chuan Xiong (Ligusticum chuanxiong *Hort.)*, Tao Ren (Prunus persica *(L.) Batsch.), and* Hong Hua (Carthamus tinctorius *L). To treat ischemic stroke, TCM practitioners prescribe herbs that can open the blood vessels and promote blood flow in circulation. THSWT has long been employed clinically to promote blood circulation to relieve women's irregular menses disorder, and is also used to treat CVDs such as hypertension and angina [11, 12]. Furthermore, it can increase blood flow of the microcirculation thereby regulating diabetic neuropathies and glucocorticoid-induced avascular necrosis of the femoral head [13, 14].

Hitherto, research tended to report on the advantages of THSWT in treating some CVDs; however, there is no systematic evaluation of the treatment of ischemic stroke. Hence, this study investigated the therapeutic milieu of THSWT in MCAO-induced cerebral infarction, and the findings were utilized to further characterize the neuroprotective potential of THSWT.

## 2. Methods

### 2.1. MCAO-Induced Transient Focal Cerebral Ischemia in Rats

Male Wistar rats (250~300 g) were used in this study. All animal experiments and care were performed according to the *National Research Council Guide for the Care and Use of Laboratory Animals* and was approved by the Institutional Animal Care and Use Committee (IACUC) of Taipei Medical University (no. LAC-98-0088). Before undergoing the experimental procedures, all animals were clinically normal, were free of apparent infection or inflammation and exhibit no neurological deficits which were evaluated by spontaneous rotational test.

Animals were anesthetized with a mixture of 75% air and 25% O_2_ gases containing 3% isoflurane. The rectal temperature was maintained at 37 ± 0.5°C. The right middle cerebral artery (MCA) was occluded as described in our previous report [15]. Briefly, the right common carotid artery (CCA) was exposed, and a 4–0 monofilament nylon thread (25 mm) coated with silicon was then inserted from the external into the internal carotid artery until the tip occluded the origin of the MCA. After closure of the operative sites, the animals were allowed to wake from the anesthesia. During another brief period of anesthesia, the filament was gently removed after 1 h of MCAO. An observer blinded to the identity of the groups assessed the neurological deficits at 1 and 24 h after reperfusion (before being euthanized) by forelimb akinesia (also called the postural tail-hang) test whereas a spontaneous rotational test was used as a criterion for evaluating the ischemic insult [16]. Animals not showing behavioral deficits at the above time points after reperfusion were excluded from the study. On the other hand, reperfusion was also ensured by an improvement in ipsilateral local blood flow to at least 60% of the baseline following an initial sharp decrease to about 50% ~ 60% of the baseline caused by MCAO as determined using a continuous laser Doppler flowmeter (LDF; Oxford Array, Oxford Optronix, Oxford, UK) with a standard needle probe (pp-051).

Rats were euthanized by decapitation after 24 h of reperfusion. The brains were cut into 2-mm coronal slices starting 1 mm from the frontal pole. Each stained brain (2% 2,3,5-triphenyltetrazolium; TTC) slice was drawn using a computerized image analyzer (Image-Pro plus). The calculated infarct areas were then compiled to obtain the infarct volume (mm^3^) per brain. Infarct volumes were expressed as a percentage of the contralateral hemisphere volume using the formula: (the area of the intact contralateral [left] hemisphere—the area of the intact region of the ipsilateral [right] hemisphere) to compensate for edema formation in the ipsilateral hemisphere [15].

All animals were divided into three groups: (1) a sham-operated group (2) a group orally treated with an isovolumetric solvent (distilled water) and (3) groups orally treated with 0.7 and 1.4 g kg^−1^day^−1^ THSWT for 14 days, respectively. The THSWT was obtained from the Sun Ten Pharm. CO. (Taichung, Taiwan). Its composition and purity is as follows: Shu Di Huang 5.0 g, Bai Shao 5.0 g, Dang Gui 5.0 g, Chuan Xiong 2.5 g, Tao Ren 5.0 g, and Hong Hua 2.5 g. The above herbal mixtures (25 g) yield an amount of dry extract 7.0 g (25.0 : 7.0 = 3.6 : 1). Each 12 g contains 7 g of herbal extract and 5 g of corn starch.

### 2.2. Neurobehavioral Test

The sensorimotor integrity was conducted to assess the neurobehavior at 24 h after MCAO in rats [16]. Five categories of motor neurological findings were scored: 0, no observable deficit; 1, forelimb flexion; 2, forelimb flexion and decreased resistance to lateral push; 3, forelimb flexion, decreased resistance to lateral push, and unilateral circling; 4, forelimb flexion, unable or difficult to ambulate.

#### 2.2.1. Determination of the Expressions of Hypoxia-Inducible Factor (HIF)-1*α*, Inducible Nitric Oxide Synthase (iNOS), Tumor Necrosis Factor (TNF)-*α*, and Active Caspase-3 in MCAO-Insulted Brains

Expressions of HIF-1*α*, iNOS, TNF-*α*, and active caspase-3 in the brain at 24 h after MCAO-reperfusion injury were analyzed by immunoblotting as described by Rodrigo et al. [17] with minor modifications. MCAO-insulted and sham-operated rats were anesthetized with chloral hydrate (400 mg kg^−1^, i.p.), then the apex of the heart was penetrated with a perfusion cannula inserted through the left ventricle into the ascending aorta. Perfusion with ice-cold phosphate-buffered saline (PBS) was performed, and an incision was made in the right atrium for venous drainage. Brains were freshly removed and sectioned coronally into four sequential parts from the frontal lobe to the occipital lobe. The third of four parts of the right hemisphere was separately collected, snap-frozen in liquid nitrogen, and stored at −70°C. The frozen tissues were placed in homogenate buffer and homogenized, then sonicated for 10 s three times at 4°C. The sonicates were subjected to centrifugation (10,000× g).

The supernatant (50 *μ*g protein) was subjected to sodium dodecylsulfate polyacrylamide gel electrophoresis (SDS-PAGE) and electrophoretically transferred to polyvinylidene difluoride (PVDF) membranes (0.45 *μ*m, Hybond-P, Amersham). After incubation in blocking buffer and being washed three times with TBST buffer (10 mM Tris-base, 100 mM NaCl, and 0.1% Tween 20; pH 7.5), the blots were treated with an anti-HIF-1*α* polyclonal antibody (pAb, 1 : 1000; R&D, Minneapolis, MN), an anti-iNOS monoclonal antibody (mAb; 1 : 3000, BD Biosciences, San Jose, CA), an anti-TNF-*α* pAb (1 : 1000; Cell Signaling, Beverly, MA), and an anti-active caspase-3 pAb (1 : 250; Biovision, Mountain View, CA), or an anti-*α*-tubulin mAb (1 : 2000; Santa Cruz Biotech, Santa Cruz, CA) in TBST buffer overnight. Blots were subsequently washed with TBST and incubated with a secondary horseradish peroxidase-conjugated goat antimouse mAb or donkey anti-rabbit immunoglobulin G (IgG) (Amersham) for 1 h. Blots were then washed, and the immunoreactive protein was detected using film exposed to enhanced chemiluminescence detection reagents (ECL^+^ system; Amersham). The bar graph depicts the ratios of semiquantitative results obtained by scanning reactive bands and quantifying the optical density using Videodensitometry (Bio-1D vers. 99 image software).

### 2.3. Platelet Aggregation

Human platelet suspensions were prepared as previously described [18]. This study was approved by the Institutional Review Board (IRB) of Taipei Medical University (no. P960312) and conformed to the principles outlined in the *Helsinki Declaration*, and all human volunteers provided informed consent. In brief, blood was collected from healthy human volunteers who had taken no medicine during the preceding 2 weeks, and was mixed with acid/citrate/glucose (9 : 1 : 1, v  v^−1^). After centrifugation, the supernatant (platelet-rich plasma; PRP) was supplemented with prostaglandin E_1_ (0.5 *μ*M) and heparin (6.4 IUmL^−1^) and centrifuged for 10 min. The washed platelets were finally suspended in Tyrode's solution containing BSA (3.5 mg mL^−1^) and adjusted to about 4.5 × 10^8^ platelets mL^−1^. The final concentration of Ca^2+^ in Tyrode's solution was 1 mM. A turbidimetric method was applied to measure platelet aggregation [18], using a Lumi-Aggregometer (Payton, Scarborough, Ontario, Canada). Washed platelet suspensions (3.6 × 10^8^ platelets mL^−1^) were preincubated with various concentrations of THSWT or an isovolumetric solvent control (Tyrode's solution) for 3 min before the addition of agonists. The reaction was allowed to proceed for 6 min, and the extent of aggregation was expressed in light-transmission units.

### 2.4. Fluorescein Sodium-Induced Platelet Thrombi in Mesenteric Microvessels of Mice

This study conformed to the* Guide for the Care and Use of Laboratory Animals* (NIH Publication no. 85–23, 1996). As described previously [15], mice were anesthetized, and an external jugular vein was cannulated with PE-10 for administration of the dye and drugs. A segment of the small intestine was placed onto a transparent culture dish for microscopic observation. Venules (30~40 *μ*m) were selected for irradiation to produce a microthrombus. Filtered light from which wavelengths below 520 nm had been eliminated was used to irradiate a microvessel. Two doses of THSWT (3.2 and 16 mg kg^−1^) were administered 1 min after fluorescein sodium (15 *μ*g kg^−1^) had been given. The time lapse for inducing thrombus formation leading to cessation of blood flow was measured.

### 2.5. Electron Spin Resonance (ESR) Spectrometry

ESR spectra were recorded on a Bruker EMX ESR spectrometer using a quartz flat cell designed for aqueous solutions. Conditions of ESR spectrometry were as follows: 3456 ± 50 G power of 0.635 Mw, a modulation frequency of 100 kHz; a frequency of 9.663 GHz; a modulation amplitude of 1 G; receiver gain of 6.3 × 10^−4^, a time constant of 81.92 ms, and a conversion time of 327.68 ms. The ESR spectrum was obtained in the H_2_O_2_/NaOH/DMSO system as previously described [19]. Briefly, 100 *μ*L of DMSO and the same volumes of 25 mM NaOH and THSWT (20 and 40 *μ*g mL^−1^) were mixed in a test tube, followed by the addition of 10 *μ*L DMPO and 100 *μ*L of 30% H_2_O_2_. The reaction mixture was aspirated into a quartz flat cell and set in the ESR apparatus; scanning began 1 min after all reagents were mixed. The rate of free radical-scavenging activity was defined by the following equation: inhibition rate = 1‒[signal height (THSWT) signal height^−1^ (solvent control)] [19].

### 2.6. Data Analysis

Experimental results are expressed as the means ± S.E.M. and are accompanied by the number of observations. Paired Student's *t*-test was used to determine significant differences in the *in vivo *study of platelet plug formation. The other experiments were assessed by the method of analysis of variance (ANOVA). If this analysis indicated significant differences among the group means, then each group was compared using the Newman-Keuls method. A *P* value of <.05 was considered statistically significant.

## 3. Results

### 3.1. Reduction of MCAO-Induced Focal Cerebral Ischemia by THSWT in Rats

**Figure 1:**
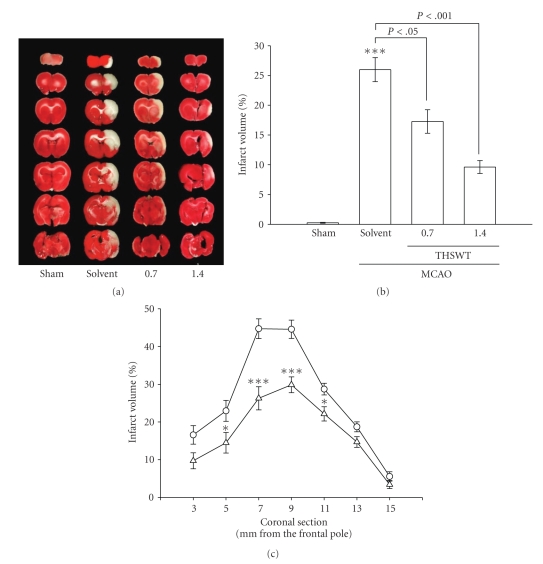
Effects of Tao-Hong-Si-Wu-Tang (THSWT) in middle cerebral artery occlusion-(MCAO-) induced focal cerebral ischemia in rats. (a) Coronal sections of 2,3,5-triphenyltetrazolium-(TTC-) stained brains, (b) dose-response effect of THSWT, and (c) the infarct area at various distances from the frontal pole 24 h after MACO-reperfusion rats. Cerebral infarction in sham-operated (sham) or MACO-reperfusion rats is from a representative animal that received the solvent (distilled water) or THSWT (0.7 and 1.4 g kg^−1^day^−1^) orally for 14 days. (a) TTC-stained brains and (b) infarct volumes were calculated as described in “Methods”, and data are presented as the infarct volume for each animal in the group as well as the means ± S.E.M. (*n* = 7). ***P < .001, compared to the sham-operated group. (c) Forebrain profiles of the infarct area at various distances from the frontal pole as described in “Methods”. Each point (○, solvent-treated group; Δ, THSWT 0.7 g kg^−1^day^−1^-treated group) and vertical bar represent the means ± S.E.M. (*n* = 7). *P < .05 and ***P < .001, compared to the solvent-treated group.

All animals in this study showed similar physiological values (i.e., rectal temperature and mean arterial blood pressure) before, during, and after MCAO among the groups (data not shown). Neither abnormal behavior, depression of respiration, nor hypothermia was observed in the solvent- or THSWT-treated groups. The cerebral infarction was examined using 2-mm-thick slices of the cerebrum 24 h after MCAO reperfusion in rats through TTC staining.  Figure 1(a) shows typical photographs of coronal sections of the sham-operated, solvent-(distilled water) treated, and THSWT-treated groups (0.7 and 1.4 g kg^−1^day^−1^) prior to the ischemic insult. Administration of THSWT at 0.7 and 1.4 g kg^−1^day^−1^ showed dose-dependent reductions in infarct volume (white area) compared to the solvent-treated group (solvent, 23.4% ± 0.6% versus 0.7 g kg^−1^day^−1^, 18.9% ± 0.8%; 1.4 g kg^−1^day^−1^, 8.2% ± 0.3%, *n* = 7) (Figure 1(b)).  Figure 1(c) gives statistical results of the infarct areas of solvent- and THSWT-(0.7 g kg^−1^day^−1^) treated groups at various distances from the frontal pole. The infarct area was largest between the 3rd and 4th sections in both groups. Oral administration of THSWT (0.7 g kg^−1^day^−1^) markedly reduced the infarct area in all regions, especially in sections three to five (Figure 1(c)). In addition, an obvious improvement was observed in neurological function of THSWT-(0.7 g kg^−1^day^−1^) treated rats at 24 h after MCAO than that of solvent-treated group (1.3 ± 0.4 versus 3.2 ± 0.7, *n* = 7; *P* < .05).

### 3.2. Inhibition of HIF-1*α*, iNOS, TNF-*α*, and Active Caspase-3 Expressions by THSWT in Ischemic Cerebral Tissues

**Figure 2:**
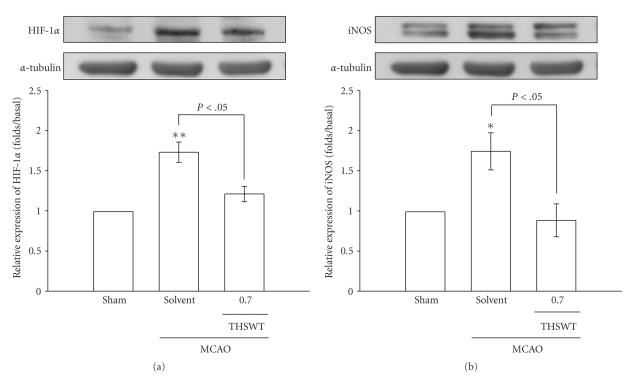
Effects of Tao-Hong-Si-Wu-Tang (THSWT) on the expressions of (a) hypoxia-inducible factor (HIF-1*α* and (b) inducible nitric oxide synthase (iNOS) in cerebral homogenates 24 h after middle cerebral artery occlusion-(MCAO-) reperfusion injury in rats. Fresh brains from sham-operated (lane 1), solvent-treated (lane 2), and THSWT- (0.7 g kg^−1^day^−1^) treated (lane 3) rats were removed and sectioned coronally into four sequential parts from the frontal lobe to the occipital lobe. The third of four sequential parts of the ischemic-injured hemisphere was separately collected, homogenized, and centrifuged. The supernatant (50 *μ*g protein) was then subjected to SDS-PAGE, and transferred onto membranes for analysis of HIF-1*α* and iNOS expressions. The results are representative examples of three similar experiments. Data are presented as the means ± S.E.M. *P < .05 and **P < .01, compared to the sham-operated group (lane 1). Equal loading in each lane is demonstrated by similar intensities of *α*-tubulin.

**Figure 3:**
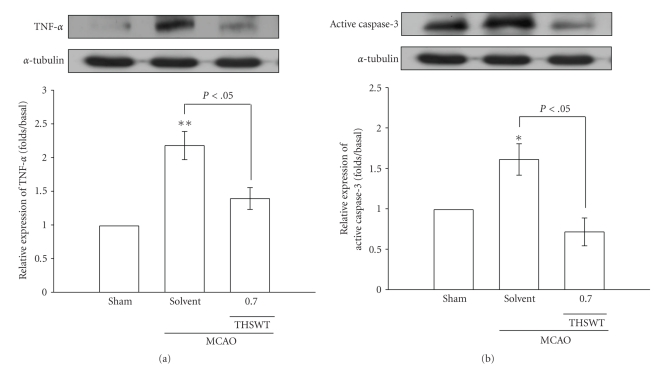
Effects of Tao-Hong-Si-Wu-Tang (THSWT) on the expressions of (a) tumor necrosis factor (TNF)-*α* and (b) active caspase-3 in cerebral homogenates 24 h after middle cerebral artery occlusion-(MCAO-) reperfusion injury in rats. Fresh brains from sham-operated (lane 1), solvent-treated (lane 2), and THSWT (0.7 g kg^−1^day^−1^)-treated (lane 3) rats were removed and sectioned coronally into four sequential parts from the frontal lobe to the occipital lobe. The third of four sequential parts of the ischemic-injured hemisphere was separately collected, homogenized, and centrifuged. The supernatant (50 *μ*g protein) was then subjected to SDS-PAGE and transferred onto membranes for analysis of TNF-*α* and active caspase-3 expressions. The results are representative examples of three similar experiments. Data are presented as the means ± S.E.M. *P < .05 and **P < .01, compared to the sham-operated group (lane 1). Equal loading in each lane is demonstrated by similar intensities of *α*-tubulin.

Results of immunoblotting of MCAO-insulted cerebral tissues are shown in Figures 2 and 3. As shown in Figure 2(a), HIF-1*α*, detected as a major band of approximately 120 kDa 24 h after MCAO-reperfusion injury (lane 2), was more pronounced than that of levels obtained in the corresponding area of the sham-operated group (lane 1). THSWT (0.7 g kg^−1^day^−1^) treatment significantly (*P* < .05) suppressed the expression of HIF-1*α* in ischemic cerebral tissues (Figure 2(a), lane 3). In Figure 2(b), the iNOS band, detected as a major band of approximately 135 kDa, showed significant increases in ischemic cerebral tissues 24 h after MCAO-reperfusion compared to that of sham-operated rats. With oral administration of THSWT (0.7 g kg ^−1^day^−1^), iNOS expression was markedly reduced in MCAO-reperfused rats (Figure 2(b)).

In addition, negative immunostaining was obtained for TNF-*α* in the sham-operated group (Figure 3(a), lane 1). At 24 h after MCAO-reperfusion, strong staining of TNF-*α* was observed in ischemic cerebral tissues (lane 2) compared to levels obtained in the corresponding area of the sham-operated group (lane 1). Again, THSWT (0.7 g kg^−1^day^−1^) obviously abolished the elevation of TNF-*α* (Figure 3(a), lane 3). Transient MCAO resulted in a significant increase in the expression of active caspase-3 (17 kDa) in the injured hemisphere compared to levels obtained in the corresponding area of the sham-operated group (Figure 3(b), lane 2). THSWT (0.7 g kg^−1^day^−1^) treatment markedly reduced this reaction (Figure 3(b), lane 3).

### 3.3. Effects of THSWT on Platelet Aggregation In Vitro and Thrombus Formation in Microvessels of Fluorescein Sodium-Pretreated Mice In Vivo

**Figure 4:**
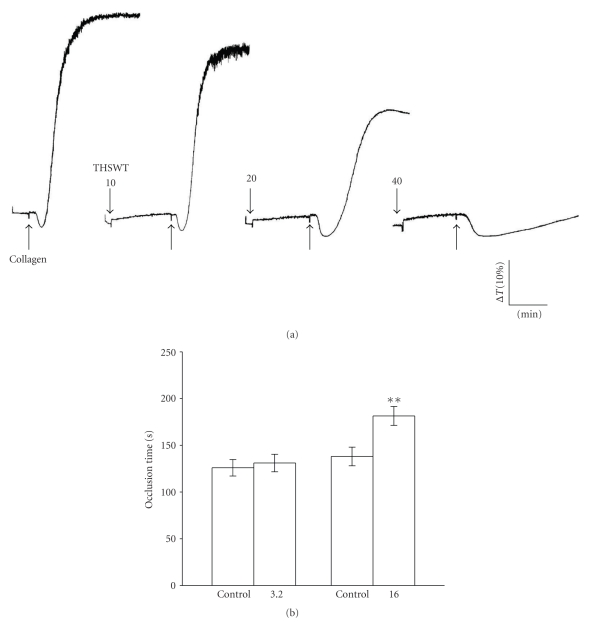
Inhibitory effect of Tao-Hong-Si-Wu-Tang (THSWT) on platelet aggregation *in vitro* and prolongation of the occlusion time for inducing thrombus formation *in vivo*. (a) Washed human platelets (3.6 × 10^8^ platelets mL^−1^) were preincubated with THSWT (20 and 40 *μ*g ml^−1^) for 3 min, followed by the addition of collagen (1 *μ*g mL^−1^) to trigger platelet aggregation. The profiles are a representative example of three similar experiments. (b) Mice were administered the solvent control (normal saline) or THSWT (3.2 and 16 g kg^−1^), after which mesenteric venules were selected for irradiation to induce microthrombus formation. Data of the bar graphs in (b) are presented as the means ± S.E.M. of the occlusion time (s) for inducing platelet plug formation (*n* = 4). **P < .01, compared to the individual solvent control group.

Collagen (1 *μ*g mL^−1^) triggers a more-pronounced platelet aggregation in washed platelet suspensions (Figure 4(a)). THSWT (10~40 *μ*g mL^−1^) concentration-dependently inhibited platelet aggregation stimulated by collagen (1 *μ*g mL^−1^). At *4* *0* *μ*g mL^−1^, THSWT almost inhibited platelet aggregation stimulated by collagen (1 *μ*g mL^−1^) in washed human platelets (Figure 4(a)). The solvent control (Tyrode's solution) did not significantly affect platelet aggregation in this reaction (data not shown). For the study of thrombus formation in microvessels of fluorescein sodium (15 *μ*g kg^−1^)-pretreated mice, the time to occlusion was approximately 140 s. When THSWT was administered at 16 g kg^−1^ after pretreatment with fluorescein sodium, occlusion times were markedly prolonged compared to the solvent controls (normal saline, 139.3 ± 10.0 s versus 16 g kg^−1^, 182.7 ± 10.1 s, *n* = 4, *P* < .01) (Figure 4(b)).

### 3.4. Inhibition of Hydroxyl Radical (OH^●^) Formation by THSWT in ESR Spectrometry

**Figure 5:**
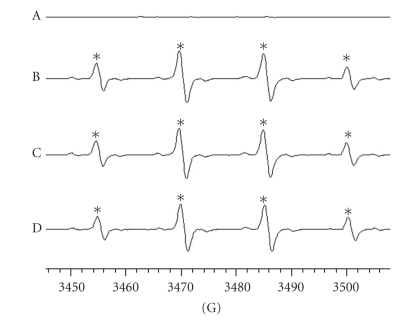
Inhibition of the free radical-scavenging activity by Tao-Hong-Si-Wu-Tang (THSWT) in the H_2_O_2_/NaOH/DMSO system. The signal of hydroxyl radical peaks was observed in electron spin resonance (ESR) experiments. (a) Resting spectrum (without H_2_O_2_), (b) typical ESR spectra in the presence of solvent control (distilled water), (c) THSWT (20 *μ*g mL^−1^), and (d) (40 *μ*g ml^−1^) in the H_2_O_2_/NaOH/DMSO system. The spectrum is a representative example of four similar experiments*.* An asterisk (*) indicates formation of hydroxyl radicals.

In this study, typical ESR signals of hydroxyl radicals (OH^●^) were observed as shown in Figure 5. THSWT (20 and 40 *μ*g mL^−1^) did not significantly suppress hydroxyl radical formation compared to the solvent-treated group. This observation provides direct evidence suggesting that the neuroprotective effect of THSWT was not mediated by free radical-scavenging activity.

## 4. Discussion

This study demonstrates for the first time that THSWT possesses neuroprotective activity against MCAO-induced cerebral infarction in rats. Cerebral ischemia restricted to the distribution of the MCAO gives rise to focal metabolic disturbances that result in infarction, neuronal necrosis, and brain edema [20]. MCAO-reperfusion injury induces HIF-1*α*, iNOS, TNF-*α*, and active caspase-3 expressions, which may represent the response of neurons suffering from ischemic insult. Animal models of focal cerebral ischemia, for which MCAO is usually used, reproduce the pattern of ischemic brain damage observed in many human ischemic stroke patients [21].

**Figure 6:**
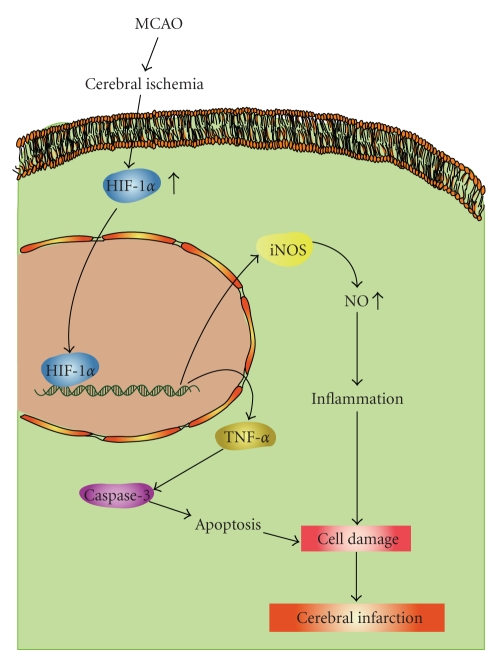
Hypothetical scheme of middle cerebral artery occlusion-(MCAO-) induced focal cerebral ischemia. MCAO decreases the blood flow of ischemic area followed by inducing a release of transcription factor HIF-1*α* . HIF-1*α* translocates into the nucleus in activating genes of iNOS and TNF-*α*. iNOS may contribute to enhance the inflammatory responses by elevating nitric oxide (NO). TNF-*α*, it shows to be involved in apoptosis through the activation of downstream caspases-3, and subsequently induces cerebral infarction.

The increased HIF-1*α* protein level observed after MCAO-reperfusion is presumably induced by a loss of the oxygen supply [21], resulting in a greater extent of binding activity to the iNOS gene which produces a consequent peak of iNOS protein expression. Since the iNOS gene contains the hypoxia-responsive enhancer (HRE) sequence to which HIF-1*α* binds [22], results from primary neuronal cultures of cells demonstrated that HIF-1*α* binds to the iNOS promoter gene under hypoxic conditions. Such binding is associated with an increase in iNOS expression [23] (Figure 6). Furthermore, HIF-1*α* combining with p53 may promote apoptotic cell death in ischemic areas [22]. In addition, the increased expression of iNOS may also contribute to enhanced neuronal injury, since iNOS knockout mice show reduced brain damage after ischemia [24].

Several apoptosis-related proteins, including caspases-9 and -3, were all strongly expressed after ischemic injury. In addition, hypoxia may cause HIF-1*α* to bind to p53 in order to stabilize it, and also activates the expression of various genes including Bax (a proapoptotic member of the Bcl-2 family) [25]. Bax is translocated to mitochondria where it releases cytochrome c into the cytosol to interact with Apaf-1 to activate caspase-9, which in turn activates downstream caspases, such as active caspase-3 [26]. In the present study, we showed that elevations of active caspase-3 and iNOS expressions occurred in the same time frame as HIF-1*α* expression after ischemic injury, and these expressions could be significantly suppressed by THSWT (Figure 6). In addition, TNF-*α* is one of the key immunomodulatory and proinflammatory cytokines upregulated during brain ischemia [27]. Administration of TNF-*α* during ischemic brain insult was shown to augment injury, as evidenced by increased tissue damage and neurological deficits [28]. In addition to inflammation, TNF-*α* was also shown to be involved in apoptosis [28]. In this study, we demonstrated that THSWT can inhibit TNF-*α* expression during brain ischemia. Therefore, inhibition of active caspase-3 expression by THSWT may occur, at least partially, through the inhibition of TNF-*α* expression in ischemic brain injury (Figure 6).

Platelet aggregation plays a pathophysiological role in cerebrovascular disorders. Inhibition of platelet aggregation by drugs may represent an increased therapeutic possibility for such diseases. We previously demonstrated that endothelial cell injury induces platelet aggregation and adhesion to vessel walls [29]. In the present study, we found that THSWT inhibited both platelet aggregation *in vitro* and thrombosis* in vivo*. In the thrombotic study, the mesenteric venules were continuously irradiated by fluorescein sodium throughout the entire experimental period, thus leading to strong damage to endothelial cells as previously described [30]. Therefore, the dose (16 g kg^−1^) of THSWT employed in this model was relatively higher than that (0.7 g kg^−1^day^−1^) in MCAO-induced cerebral ischemia. Furthermore, we also examined whether THSWT has direct free radical-scavenging activity in a cell-free system. In this study, the mechanisms of free radical formation in the H_2_O_2_/NaOH/DMSO system were assumed to be from superoxide anions and hydroxyl radicals being generated from the degradation of hydrogen peroxide [19]. The superoxide anion changes into a hydroxyl radical by the catalytic action of contaminating trace iron, so that the amount of hydroxyl radicals is consequently relatively larger than that of superoxide anions. Using this system, the free radical-scavenging activity of hydroxyl radicals could be evaluated. In this study, THSWT did not significantly inhibit hydroxyl radical formation *in vitro*. Thus, the neuroprotection of THSWT might not involve, at least partly, the inhibition of free radical formation.

In conclusion, the most important findings of this study suggest that the neuroprotective effect of THSWT on cerebral ischemic damage in MCAO-reperfusion rats is probably mediated by the inhibition of HIF-1*α* and TNF-*α*, followed by the inhibition of inflammatory responses (i.e., iNOS), apoptosis (active caspase-3), and platelet activation. The rationale for the use of THSWT is based on the fact that multiple deleterious processes in different cell types of organelles are initiated during ischemia-reperfusion injury which ultimately synergistically moves toward irreversible injury. Therefore, treatment with THSWT is not limited to one factor but involves many mechanisms, most of which may be interrelated. For example, THSWT-induced neuroprotection is related to inflammation, NO, and apoptosis, and many of those factors (such as iNOS, active caspase-3, etc.) are related to HIF-1*α*. We speculate that the suppression of these molecules and morphological changes may lead to improvements in patients with ischemic stroke. Thus, these results provide scientific validation of a better understanding of the effectiveness of THSWT in ischemia-reperfusion brain injury and related diseases.

## 5. Acknowledgment

This work was supported by Grants from the National Science Council of Taiwan (nos. NSC97-2320-B-038-016-MY3 and NSC 95-2320-B-195-003-MY2) the Committee on Chinese Medicine and Pharmacy (no. CCMP97-RD-008) Mackay Memorial Hospital Medical Research Fund (no. MMH9828) and Wan-Fang Hospital-Taipei Medical University(no. 97TMU-WFH-01). Chih-Jen Wu, Jui-Tai Chen contributed equally to this work.
